# Bone mineral density as potential individual prognostic biomarker in patients with neurosurgically treated spinal metastasis

**DOI:** 10.1007/s00432-025-06142-9

**Published:** 2025-03-10

**Authors:** H. Asoglu, T. Lampmann, M. Jaber, L. Khalafov, J. Dittmer, I. Ilic, G. H. Gielen, M. Toma, H. Vatter, Z. Bendella, M. Schneider, C. Schmeel, M. Hamed, M. Banat

**Affiliations:** 1 Department of Neurosurgery, University Hospital Bonn, Bonn / Venusberg-Campus 1, 53127 Bonn, Germany; 2 Department of Neuroradiology, University Hospital Bonn, Bonn, Germany; 3 Department of Neuropathology, University Hospital Bonn, Bonn, Germany; 4 Department of Pathology, University Hospital Bonn, Bonn, Germany

**Keywords:** Spinal metastasis, Bone mineral density, Overall survival

## Abstract

**Introduction:**

Bone mineral density (BMD) plays a crucial role in diagnosing and treating various systemic chronic diseases. Patients with multiple or singular spinal metastasis (SM) are typically in advanced stages of systemic cancer, often leading to significant alterations in BMD. The present study investigated the prognostic value of perioperative Hounsfield units (HU) as a surrogate independent marker for estimated BMD in patients with SM after surgical treatment (ST).

**Methods:**

HU values, serving as a surrogate for estimated BMD, were measured from circular regions of interest (ROIs) in the spine -first lumbar vertebra (L1)- from routine preoperative staging computed tomography (CT) scans in 187 patients after ST. The estimated BMD was stratified into pathologic and physiologic values and correlated with survival parameters in our cohorts.

**Results:**

Median L1 BMD of 92 patients (49%) with pathologic BMD was 79.5 HU (IQR 67.25–93.5) compared to 145 HU (IQR 123–166) for 95 patients (51%) with physiologic BMD (*p* ≤ 0.001). Patients with pathological BMD exhibited a median overall survival of 8 months compared to 12.2 months in patients with physiologic BMD (*p* = 0.006). Multivariable analysis revealed pathologic BMD as an independent negative prognostic predictor for increased 1 year mortality (AUC: 0.637, 95% CI: 0.556–0.718; *p* = 0.001).

**Conclusions:**

The present study demonstrates that decreased perioperative BMD values, as derived from HU measurements, may represent a previously unrecognized negative prognostic factor in patients of SM after ST. The estimated perioperative BMD could emerge as an individualized, readily available potential biomarker for prognostic, treatment, and discussion of affected patients with SM.

## Introduction

Systemic acute or chronic tumor disease with singular or multiple spinal metastases (SM) has an increasingly prominent role in the daily clinical practice of spine surgeons and in the lives of affected cohort with tumor patients (Coleman 2006; Brande et al. 2022). It is estimated that approximately 5 − 15% of all systemic cancer patients will ultimately develop singular or multiple SM (Brande et al. 2022; Jenis et al. 1999; Jacobs and Perrin 2001). Among the primary culprits are lung cancer, prostate cancer and breast cancer with the primary tumor remaining elusive in 2–3% to10% of cases (Greenlee et al. 2000; Ulmar et al. 2007). Surgery stands as a common and established treatment modality and option (Furlan et al. 2022). In addition to the liver and lungs, the skeletal system and bones are also common sites for metastatic spread (Macedo et al. 2017; Maccauro et al. 2011). Surgical alternative options for treating SM encompass a varied spectrum, from transpedicular biopsy coupled with kyphoplasty or vertebroplasty (Stangenberg et al. 2017; Georgy 2010), to isolated decompression of spinal canal (Patchell et al. 2005), or in addition with minimally invasive percutaneous surgical procedures (Miscusi et al. 2015) and open -short or complex- instrumentation with augmented screws implantation (Ringel et al. 2017; Park et al. 2019a, b), at times necessitating anterior-posterior stabilization with fusion (Ulmar et al. 2006; Gezercan et al. 2016).

The overall goal of surgical intervention is the mitigation or prevention of neurological deficits, combined with a focus on enhancing the patient’s quality of life (Fehlings et al. 2016; Depreitere et al. 2020). Additionally, surgical treatment serves as a tool to attain a histo-pathological diagnosis of the spinal tumor manifestation and potentially improves overall survival (OS) (Patchell et al. 2005; Krober et al. 2004).

Our study group at our neuro-oncological and spine center is concerned with the clinical parameters of patients with surgical treated spinal metastases, which can have a positive or negative influence on the course of treatment and OS of Patients with SM (Hamed et al. 2022a, b; Banat et al. 2024). Another important clinical prognostic value in the treatment of tumor patients with systemic metastasis is the bone mineral density (BMD), which has already been established and recently been described as another potentially valuable imaging marker in the literature for chronic or acute diseases or some tumor entities, especially for patients with brain metastasis (Schulze-Hagen et al. 2021; Yao et al. 2019; Qu et al. 2013; Sharshar et al. 2020; Ilic et al. 2022). In our center, BMD is used in cranial metastases, which we have also published (Ilic et al. 2022).

Therefore, the aim of this study was to evaluate the potential prognostic value of quantitatively assessed BMD in perioperative CT scans of patients with SM after neurosurgical treatment.

## Methods

### Patients selection and inclusion criteria

A retrospective cohort study was conducted; all patients aged > 18 years who had undergone surgical treatment for SM between 2015 and 2020 at our neurosurgical department of the University Hospital Bonn. Comprehensive clinical and neurological data, including age, gender, primary tumor type, SM location of the spine, details of the surgical procedure, the extent of affected spinal vertebrae, American Society of Anesthesiologists (ASA) score, perioperative clinical-neurological assessment, and functional status measured by the American Spinal Injury Association (ASIA) Score (Asia 2019), were recorded and documented.

The current clinical functional status was further evaluated using the Karnofsky Performance Scale (KPS) upon admission, categorizing patients into KPS ≥ 70% or KPS < 70%, as previously described (Schuss et al. 2021; Hamed et al. 2023; Schweppe et al. 2023; Ilic et al. 2021). The Charlson Comorbidity Index (CCI) was evaluated to quantify the comorbidity burden of patients before undergoing surgical treatment (Hamed et al. 2022a, b; Schneider et al. 2020; Lehmann et al. 2023).

Perioperative imaging of spine -the first lumbar vertebral body- was required to determine BMD.

Therefore, only patients were included with completely data, especially who had received appropriate perioperative staging computed tomography (CT; with depicting of the first lumbar vertebral bone body).

Overall survival (OS) was evaluated from the date of surgery until last observation or death as previously described (Hamed et al. 2022a, b).

Following histopathological analysis, all included patients underwent thorough assessment by our internal Neurooncological Tumor Board at the center, comprised of neurosurgeons, radiation/radiosurgery therapists, neuro-oncologists, colloquies from neuroradiology, oncology and neuro-pathology. Therapeutic Recommendations for post-surgery management were predefined through interdisciplinary consensus (Schafer et al. 2021).

**Fig. 1:**
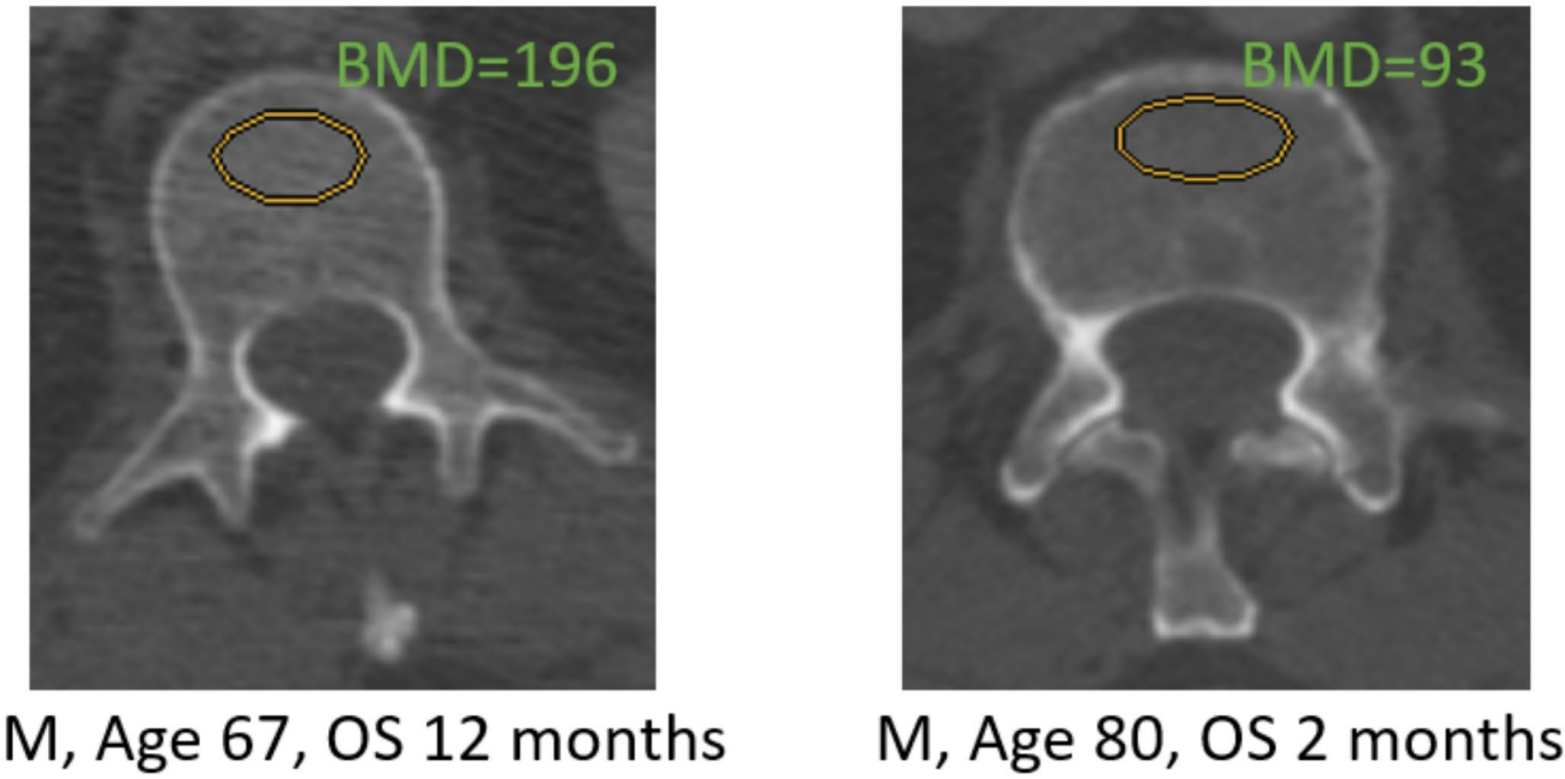
Example of BMD measurements BMD, bone mineral density

Patients were grouped into two cohorts for further analysis: those with normal BMD and those with a pathologic BMD, according to the results of Jang et al. (Jang et al. 2019). Figure 1 illustrates exemplary measurements in two patients with high and low BMD. However, in our imaging, we used only the transverse plane for L1 measurements. In the other case of a compression fracture in L1, either T12 or L2 was used for trabecular attenuation measurement. CT Image analysis and measurements were all performed using perioperative imaging archiving and communication system (ImPAX) at our center.

#### Exclusion criteria

All patients, who were classified as non-operable and those lacking complete clinical and morphological data or follow-up information were excluded from our analysis. Furthermore, patients for whom no further follow-up data regarding survival was obtainable, typically due to ongoing treatment at external healthcare institutions, were excluded. The patient was excluded if reliable L1 trabecular measurement was not possible due to focal sclerotic or focal lytic lesions, abnormalities, and/or artifacts, furthermore metastasis of L1. This means that in the case of infiltration of L1, the patient was excluded from the evaluation.

#### Study design

The study adhered to the ethical principles outlined in the 1964 Helsinki Declaration and received approval from the Ethics Committee of the University Hospital Bonn (protocol no. 067/21). Given the retrospective nature of the study, the acquisition of informed consent from participants was not pursued.

### CT image performance and analysis

CT scans were perioperative performed prior or after the surgical treatment of the SM. Standards of CT: multi-detectorscanner, constant peak voltage of 120 kV with variable protocol-specific tube current (mA) settings. Of note, kV- settings have a strong effect on bony HU values, whereas mA only affects noise levels and not HU score. A board-certified neuroradiologist measured in the lumbar spine mean L1 trabecular bone attenuation on a single axial CT image and plane by manually placing an oval region of interest at the lumbar vertebra (ROI) in trabecular space in the anterior whorl body as described by Jang et al. (Jang et al. 2019). In case of compression fracture in L1, either T12 or L2 was used for trabecular attenuation measurement. Lytic or focal sclerotic lesions were avoided.

Throughout data acquisition the neuroradiologist was blinded to all patient characteristics, treatment history and OS. We used the same oval ROI size for all datasets. Those with sclerotic lesion, abnormalities or artifacts were excluded. Other consistency tests weren`t done.

### Statistical analysis and graphical illustration

Data illustration, analysis and collection were conducted utilizing the SPSS software package for Windows (Version 27, IBM Corp., Armonk, NY). Categorical variables underwent analysis through contingency tables, employing the Fisher’s exact test when assessing two variables and the chi-square test when evaluating more than two variables. Non-normally distributed data were subjected to the Mann-Whitney U-test. Survival rate comparisons were performed utilizing the Gehan-Breslow-Wilcoxon test. Overall survival (OS) rates were assessed using the Kaplan-Meier method, with Graph Pad Prism software for MacOS (Version 9.4.1, Graph pad Software, Inc., San Diego, California, USA) employed for this purpose. To identify positive or negative predictors of elevated 1-year mortality, a multivariate logistic regression model was constructed using a backward stepwise approach. Statistical significance was determined at *p* < 0.05. The statistical analysis was based on the use of the software by the authors of the paper.

## Results

### Patient characteristics

A total of 187 patients fulfilled the inclusion requirements and were therefore selected for further statistical analysis.

The median age at admission was 65 years (IQR 57–74 years). The male gender distribution was dominant with 58.7%. Patients revealed a preoperative KPS ≥ 70 in a total of 69%. Median BMD was 113 HU (IQR 80–146 HU). The median OS for all patients with surgically treated SM was 9.73 months (range 3-19.25 months). More detailed baseline patients characteristics are shown in Table 1.

**Table 1:** Patient characteristics*

	*n* = 187
**Median age (IQR) (in yrs)**	65 (57–74)
Female sex	70 (37.4)
ASA Score ≥ 3	117 (62.6)
Primary tumor site	
Lung	41 (21.9)
Breast	21 (11.2)
Prostate	38 (20.3)
Others	87 (46.5)
Location of disease	
Cervical	17 (9.1)
Thoracic	107 (57.2)
Lumbar	30 (16.0)
Combined	33 (16.7)
Surgery	
Decompression	117 (62.6)
Stabilization	70 (37.4)
Levels of disease	
1–2	113 (60.4)
≥ 3	74 (39.6)
Median CCI (IQR)	8 (6–10)
KPS ≥ 70	129 (69.0)
Pre-operative neurological deficit (ASIA A-C)	55 (29.4)
Median OS (IQR) (in months)	9.75 (3-19.25)
1-year mortality	98 (52.4)
Median BMD (in HU)	113 (80–146)

*Values represent the number of patients unless indicated otherwise (%)

ASA, American Society of Anesthesiology physical status classification system; ASIA, American Spinal Injury Association; CCI, Charlson comorbidity index; KPS Karnophysky Performance Scale; IQR, interquartile range; n, number of patients; OS, overall survival; SM, spinal metastasis yrs, years. Values represent number of patients unless indicated otherwise (%). BMD, bone mineral density; HU, Hounsfield unit

### Diesease and patients-related characteristics dependent on pathologic and physiologig BMD-levels

Median L1 BMD of 92 patients (49%) with pathologic BMD was 79.5 (HU) (IQR 67.25–93.5) compared to 145 (IQR 123–166) for 95 patients (51%) with physiologic BMD (*p* ≤ 0.001). All common known entities of the various tumors were equally represented in both groups. Neither lung carcinoma nor prostate carcinoma nor breast carcinoma showed a statistical dominance in the univariate analysis. Further stratification according to baseline characteristics in both groups is given in Table 2.

**Table 2:** Patients with surgically treated SM stratified for pathologic and physiologic BMD (univariate analysis)

	Patients with pathologic BMD *N* = 92(*)	Patients with physiologic BMD *N* = 95(*)	*p*-value
Median BMD in HU (IQR)	79.5 (67.25–93.5)	145 (123–166)	*p* ≤ 0.001
Median age in years (IQR)	66 (58.5-73.75)	65 (53–74)	0.220
Female sex	37 (40.2)	33 (34.7)	0.454
Preoperative KPS < 70	35 (38.0)	23 (24.2)	0.057
ASA Score > 2	60 (65.2)	57 (60.0)	0.546
Primary tumor site			0.848
Lung	20 (21.7)	23 (24.2)	
Breast	12 (13.0)	9 (9.5)	
Prostate	18 (19.6)	21 (22.1)	
Other	42 (45.7)	42 (44.2)	
Location of disease			0.888
Cervical	9 (9.8)	8 (8.4)	
Thoracic	51 (55.4)	56 (58.9)	
Lumbar	14 (15.2)	16 (16.8)	
Combined	18 (19.6)	15 (15.8)	
Levels of disease			0.053
1–2	49 (53.3)	64 (67.4)	
≥ 3	43 (46.7)	31 (32.6)	
Median CCI (IQR)	8 (6–10)	8 (6–10)	0.405
Preoperative neurological deficit (ASIA A-C)	26 (28.3)	29 (30.5)	0.751
Surgery			0.546
Decompression	32 (34.8)	38 (40.0)	
Stabilization	60 (65.2)	57 (60.0)	
1-year mortality	60 (65.2)	38 (40.0)	**0.001**
Median OS in months (IQR)	8 (1.6–14)	12.2 (4–26)	**0.006**

*Values represent the number of patients unless indicated otherwise (%)

ASA, American Society of Anesthesiology physical status classification system; ASIA, American Spinal Injury Association; CCI, Charlson comorbidity index; KPS Karnophysky Performance Scale; IQR, interquartile range; n, number of patients; OS, overall survival; SM, spinal metastasis yrs, years. Values represent number of patients unless indicated otherwise (%). BMD, bone mineral density; HU, Hounsfield unit

### Influence of bone mineral density on overall survival and 1-year mortality in our cohort

**Fig. 2:**
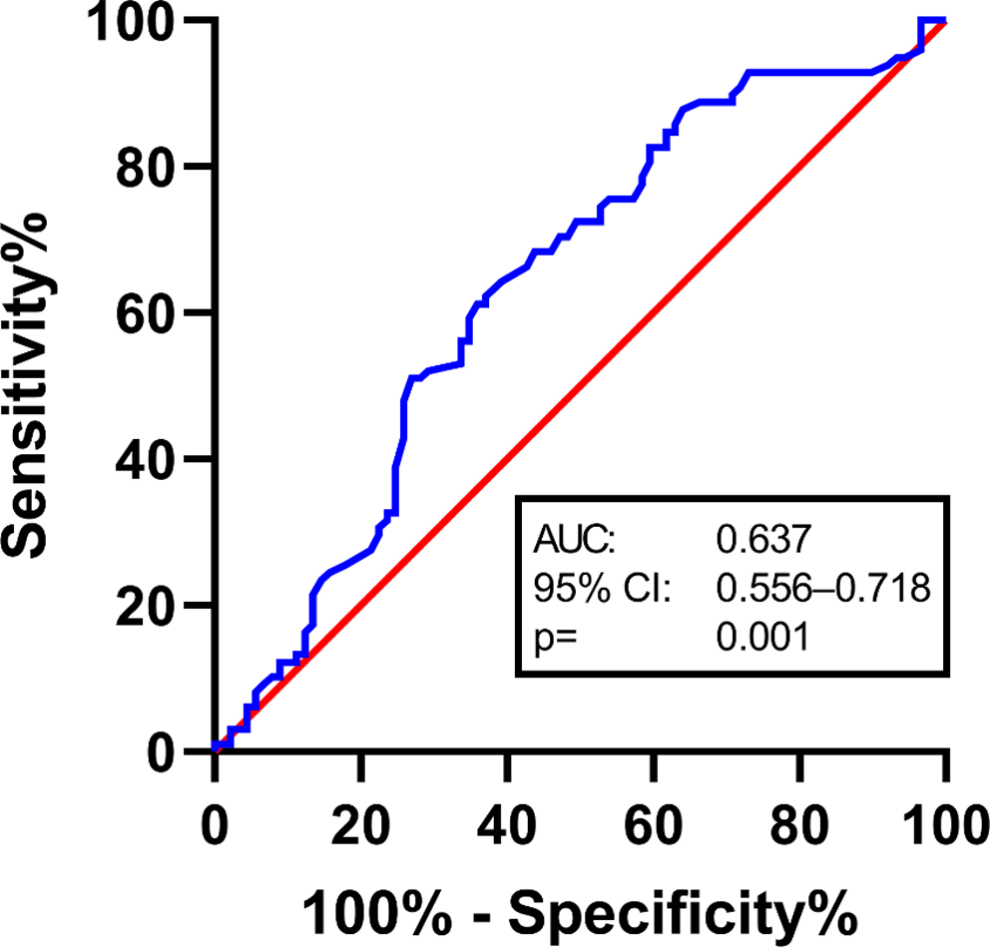
Roc analyse

The analysis of mortality was statistically significant and increased likelihood of death in relation to the group with pathologic BMD (1 year mortality rate in pathologic BMD 65.2% and in physiologic BMD group: 40.0%; p-value = 0.001). The median OS in the patients cohort with perioperative pathological BMD was 8 months compared to 12,2 months patients cohort with determined physiologic BMD (*p* = 0.006), Fig. 2.

### Multivariable analysis for predictors of 1-year mortality

We performed a roc analysis and a multivariable logistic regression analysis in order to identify positive or negative independent predictors of 1-year mortality in patients, who had undergone surgery for SM including the variables sex, preoperative KPS, preoperative CCI, the most common tumor entity, and BMD, Table 3.

**Table 3:** Multivariable regression analysis for predictors of 1-year mortality

Factors	Adjusted OR	95% CI	*p*-value
Female sex	1.86	0-91-3-79	0.090
Preoperative KPS < 70	0.68	0.27 − 0.175	**< 0.001**
CCI > 10	1.466	0.59–3.57	0.414
Tumor entity (lung)	0.641	0.28–1.47	0.293
BMD (pathologic)	2.82	1.41–5.63	**0.003**

CCI, Charlson Comorbidity Index; CI, confidence interval; KPS, Karnofsky Performance Scale; OR, Odds ratio; SM, spinal metastasis. BMD, bone mineral density; CCI, Charlson comorbidity index; HU, Hounsfield unit

**Fig. 3:**
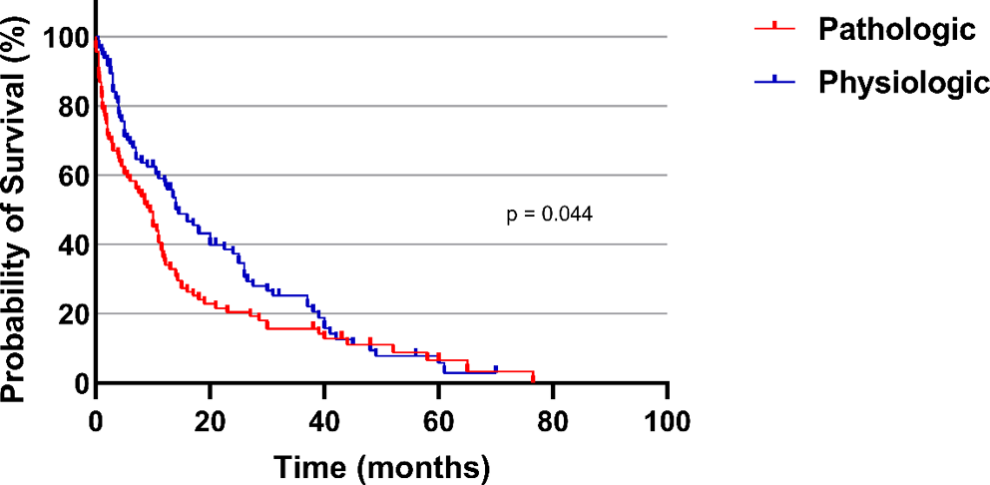
Kaplan-Meier survival analysis dependent on pathologic und physiologic SM occurrence

**Fig. 4:**
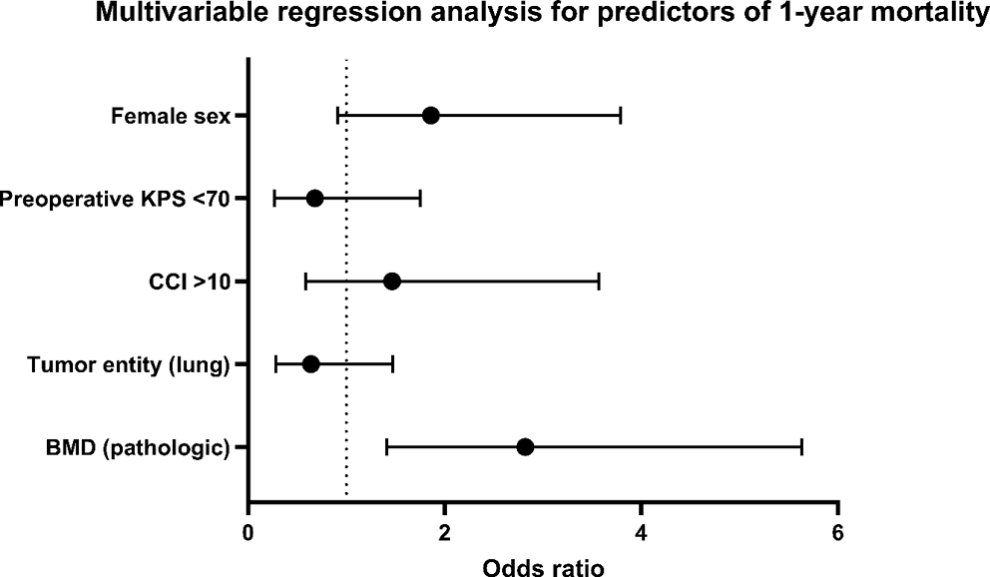
Forest plot depicting patient- and disease-related characteristics dependent on BMD in patients with surgically treated SM. CCI, Charlson comorbidity index; KPS, Karnofsky performance score; mOS, median overall survival; SM, spinal metastasis; vs., versus

The multivariable analysis identified „Preoperative KPS < 70“ (p < 0.001, OR 0.68, 95% CI 0.27 − 0.175) and „pathologic BMD” (*p* = 0.003, OR 2.82, 95% CI 1.41–5.63) as significant and independent negative predictors of 1-year mortality. The entity of tumor was not relevant. Figures 3 and 4.

## Discussion

Our research group at our spinal and neuro-oncology center focuses on clinical parameters that have a significant impact on survival in patients with spinal metastases (Hamed et al. 2022a, b; Banat et al. 2024). This retrospective single center cohort study analyzes the prognostic value of perioperative Hounsfield units (HU) as a surrogate marker for estimated BMD in patients with surgically treated SM. Our data demonstrates that decreased perioperative BMD values, as derived from HU measurements, may represent a previously unrecognized negative prognostic clinical-radiological factor in patients of SM requiring spinal neurosurgery. The best studies investigating the relationship between BMD and tumor entities are in the field of bronchial carcinomas. Prostate carcinomas metastasize to the spine very frequently, but the correlation between BMD and the influence of therapy remains very little researched, and there is much more studies investigating the effect of therapy on bone density afterwards [62–63]. Lung cancer is notably associated with the highest incidence of brain metastases (BM) and spinal metastases (SM). The occurrence of SM in lung tumor patients, as reported in the literature, ranges between 5% and 56%. This high variation is affected by various factors such as the histopatholgical type of cancer, the presence status of the epidermal growth factor receptor (EGFR) mutation/wildtype, and the actual stage of disease (Berghoff et al. 2016; Nayak et al. 2012; Goncalves et al. 2016; Wang et al. 2017; Zhang et al. 2020; Rizzoli et al. 2013). Similarly, SM is observed in 5–15% of prostate and breast cancer cases, making these pathologies among the most common to develop SM (Rizzoli et al. 2013; Hong et al. 2020; Kumar et al. 2020; Park et al. 2019a, b).

The significance of vertebral body composition imaging markers is progressively gaining importance in oncology. This diagnostic procedure is well established in specific oncological domains, although it has not yet been widely adopted in the field of spinal oncology (Troschel et al. 2020; Zhang et al. 2018; Malietzis et al. 2016). On the one hand, a major advantage of radiological imaging markers lies within the accessibility and feasibility of their kind. All oncological patients undergo staging examinations during routine screening and/or follow-up care. A variety of relevant information can be opportunistically obtained from these perioperative scans, which have an additive prognostic value for individually adapted patient care (Planchard et al. 2018).

One of the most frequently and thoroughly researched markers in the various imaging modalities in recent years is the quantification of bone, especially skeletal muscle mass. As an established and now standardized surrogate parameter for sarcopenia - a condition of pathologically reduced muscle mass - it has proven to be reliable in several oncological systemic diseases, in particular as an independent predictor of overall survival or the assessment of the response to the therapy carried out (Ilic et al. 2021; Furtner et al. 2017; Peng et al. 2021; Wong et al. 2021). For example, the prognostic significance of low muscle mass has been associated with poorer OS in patients with BM in NSCLC (Ilic et al. 2021). In addition, body physiological strength has been established as a practically relevant clinical parameter that complements quantified muscle mass well and has therefore also been investigated for temporal multimodal oncological monitoring during the course of systemic disease (Sperduto et al. 2020).

BMD constitutes another imaging marker that recently has been shown to provide valuable prognostic insights for various severe illnesses among them cardiovascular diseases and chronic lung pathologies (Qu et al. 2013; Campos-Obando et al. 2014; Trivedi and Khaw 2001). The metaanalysis of Qu et al. showed that a decrease in bone mineral density in cardiovascular diseases were significantly associated with an increased mortality. In cerebrovascular diseases like stroke the mortality was not associated with the BMD (Qu et al. 2013). The study of Schulze-Hagen et al. also detected a poor outcome in critical illness patients with decreased BMD. Patients with arterial hypertension and chronic pulmonary disease were associated with a lower BMD and an impaired short-term survival (Schulze-Hagen et al. 2021).

This retrospective single center cohort study analyzes the prognostic value of perioperative Hounsfield units (HU) as a surrogate marker for estimated BMD in patients with surgically treated SM. We found that our data demonstrates that decreased perioperative BMD values, as derived from HU measurements, may represent a previously unrecognized negative prognostic clinical-radiological factor in patients of SM requiring spinal surgery.

Known scientific information such as previous illnesses or demographic aspects were excluded as confounders, as no statistically relevant differences were found between the different groups in this pathological context. Against this background, it can be assumed that the selectivity based on bone density is obvious in the present patient clientele. Cancer patients are known to have a particularly high risk of reduced BMD and osteoporosis. In our data, bronchial cancer was most common in both groups. A large Scandinavian registry study from 2008 demonstrated that osteoporosis is associated with an increased risk of developing NSCLC in both men and women under 70 years of age (McGlynn et al. 2008). Defined exact pathophysiological mechanisms for the development of osteoporosis in bronchial carcinoma, for example, are still unknown, but it is possible to imagine that tumor-related metabolic and hormonal changes contribute in part to this association. This hypothesis is possibly confirmed by the fact that about half of the patients with non-small cell lung cancer have a positive estrogen receptor status, which may indicate that non-small cell lung cancer would interfere with these hormonal systemic circuits (Marquez-Garban et al. 2007).

In addition, it is important to emphasize that the present cohort consists of patients with SM who are at an advanced tumor stage with metastasis. In addition to the tumor, previous treatment regimens could have a significant impact on BMD values (Martinez-Alonso et al. 2014; Dreizen et al. 1990; Vassbakk-Brovold et al. 2018).

It is plausible to consider whether the observed association between bone mineral density (BMD) and reduced overall survival in both groups may have implications.

## Limitations

Our study has several limitations. While the retrospective nature of this single center cohort study harbours the risk of potential unmasked selection bias and the assessment of the L1 BMD is not yet a globally clinically accepted method for bone density measurement. BMD measurement was performed in accordance to a recently published report by Jang et al. (Jang et al. 2019), where normative values were obtained in 20,000 patients. Small patient numbers in some few subgroups may possibly interfere statistical evaluation. Also the ROI placement can vary among different analysts. We followed a strict analysis protocol to minimize those bias.

Therefore further and more multicentric studies are required to lucidate the potential prognostic impact of reduced BMD in systemic cancer patients in more detail and comprehensively, as well as to present or draw potential therapeutic aspects and conclusions. Nevertheless, our data suggest that BMD could serve as a relevant individual biomarker for the prognostic impact of cancer, the course of treatment and/or the patient-specific perioperative constitution. Due to the smaller number of cases, the patient initials should be taken into account more in the future with a larger number of patients, especially with regard to the question of whether this bias can influence the conclusion. Certain tumors also influence the BMD value in the long term; further studies will be conducted to determine whether there is a correlation in long-term results.

The evaluation of the CT images was limited to certain parameters. For more valid data analysis, several aspects should be considered in the future.

## Conclusions

The current study suggests that perioperative L1-BMD values represent a previously unrecognized potential prognostic aspect and thus a clinical imaging biomarker in patients with spinal metastasis after neurosurgical treatment. On the basis of guideline-based perioperative staging of patients with systemic metastatic tumors, BMD could prove to be an accessible surrogate biomarker for prognosis, treatment indications and clinical counseling of affected patients with singular or multiple SM.

## Data Availability

No datasets were generated or analysed during the current study.

## Abbreviations

ASA: American Society of Anesthesiologists
ASIA: American Spinal Injury Association Score
BMD: Bone mineral density
CCI: Charlson Comorbidity Index
CI: Confidence interval
CT: Computed tomography
HU: Hounsfield units
IQR: Interquartile range (25th– 75th percentile)
OS: Overall survival
ROIs: Regions of interest
SM: Spinal metastasis
ST: Surgical treatment
